# Fabrication of CaCO_3_ Microcubes and Mechanistic Study for Efficient Removal of Pb from Aqueous Solution

**DOI:** 10.3390/ma17225523

**Published:** 2024-11-12

**Authors:** Ufra Naseer, Asim Mushtaq, Muhammad Ali, Moazzam Ali, Atif Ahmad, Muhammad Yousaf, Tianxiang Yue

**Affiliations:** 1 State Key Laboratory of Resources and Environment Information System, Institute of Geographical Sciences & Natural Resources Research, Chinese Academy of Sciences, Beijing 100871, China; unaseer2018@igsnrr.ac.cn; 2 ZJU-Hangzhou Global Scientific and Technological Innovation Center, Zhejiang University, Hangzhou 311200, China; asimmushtaq@zju.edu.cn (A.M.); 12226109@zju.edu.cn (M.A.); mak.ssp@outlook.com (M.A.); 3 Department of Biological Geological and Environmental Science, University of Bologna, 40126 Bologna, Italy; atif.ahmad3@unibo.it

**Keywords:** CaCO_3_, Pb(II) contamination, adsorbent, adsorption, ion-exchange reaction

## Abstract

Pb(II) contamination in aquatic environments has adverse effects on humans even at a low concentration, so the efficient removal of Pb at a low cost is vital for achieving an environmentally friendly, sustainable, and healthy society. A variety of CaCO_3_-based functional adsorbents have been synthesized to remove Pb, but the adsorption capacity is still unsatisfactory. Herein, calcite CaCO_3_ microcubes/parallelepipeds are synthesized via simple precipitation and a hydrothermal approach and found to outperform previously reported nano-adsorbents considerably. The CaCO_3_ achieves a high removal efficiency for Pb(II) (>99%) at a very low dosage (0.04–0.1 g/L) and an initial Pb(II) concentration of 100 mg/L. The CaCO_3_ presents an excellent adsorption capacity of 4018 mg/g for Pb(II) removal and depicts good stability over a wide range of pH 6–11. The maximum adsorption kinetics are fitted well by the pseudo-second-order kinetic model, whereas the Freundlich isotherm delineates the adsorption data at equilibrium well, indicating a multilayer adsorption process. The ex situ study confirms that the Pb(II) adsorption mechanism by CaCO_3_ can be attributed to the rapid metal-ion-exchange reaction between Pb(II) and Ca^2+^. Furthermore, a red shift in the Fourier Transform Infrared (FTIR) spectroscopy peak from 1386 cm^−1^ to 1374 cm^−1^ of CaCO_3_ after Pb removal indicates the adsorption of Pb onto the surface. This adsorbent provides an opportunity to treat wastewater and can be extended to remove other toxic heavy metals.

## 1. Introduction

Lead (Pb) contamination in the environment (air, water, and soil) is a significant environmental issue because of its widespread occurrence and non-biodegradation and poses detrimental effects on both human health and ecosystems [1,2,3]. Pb contamination occurs through natural sources but is predominantly (around 98%) driven by various anthropogenic activities, such as the manufacturing of batteries, lead smelting, automotive industry, mining, vehicle exhausts, and domestic waste [4,5,6]. Pb accumulates in organisms through ingestion and inhalation, posing serious health risks, particularly in infants, young children, and pregnant women [7]. High levels of Pb can lead to kidney damage, a risk of cardiovascular disease, development delays in children (e.g., delayed speech and skills), and reproductive issues, including miscarriage and reduced fertility [8,9,10]. Therefore, the efficient extraction of Pb(II) from the environment is essential.

Extensive endeavors have been devoted to remediating environments contaminated by Pb(II), including ion exchange, chemical precipitation, electrochemical treatment, membrane exchange, bio-remediation, and adsorption [11,12,13,14]. Nevertheless, the practical application of most of the above-mentioned remediating approaches is restricted due to many protocols such as the electricity use, high reagent requirements, complex operation, inefficient removal of heavy metals, and expensive equipment [15]. As one of the widely accepted approaches for effective Pb(II) removal, the adsorption method provides some merits including inexpensiveness, simple operation, and high removal efficiency.

In the last few decades, various adsorbents have been used to extract Pb(II) from wastewater, such as carbon-based materials (graphene, porous carbon, activated carbon), metal hydroxides, biochar, polymer materials, etc. [16,17,18]. Recently, CaCO_3_ obtained from biogenic and geological sources has received considerable attention in heavy metal removal due to its low cost, high abundance, environmental friendliness, and high efficiency [19]. It exists mainly in three kinds of anhydrous crystalline forms: vaterite, aragonite, and calcite. Among these, calcites are considered more stable and less soluble in an aqueous solution [20]. A variety of CaCO_3_-based materials produced by various synthesis strategies have been used to enhance the adsorption capacity towards Pb(II) removal. For instance, a biogenic CaCO_3_ adsorbent obtained from oyster shells presented an outstanding removal capacity of 1667 mg/g for Pb(II) removal due to its hierarchical porous hybrid structure [21]. Crystal plane engineering was used to synthesize Mg-doped CaCO_3_ to further enhance the adsorption capacity (1961.9 mg/g) [2]. Ma and colleagues fabricated a hierarchical meso–microporous hybrid architecture and obtained an excellent adsorption capacity of 3242.48 mg/g [22]. Many other functional CaCO_3_ materials presented various advantages and good Pb(II) removal performance, but their adsorption capacity and removal efficiency are still unsatisfactory [1,4,23,24,25,26,27].

**Figure 1:**
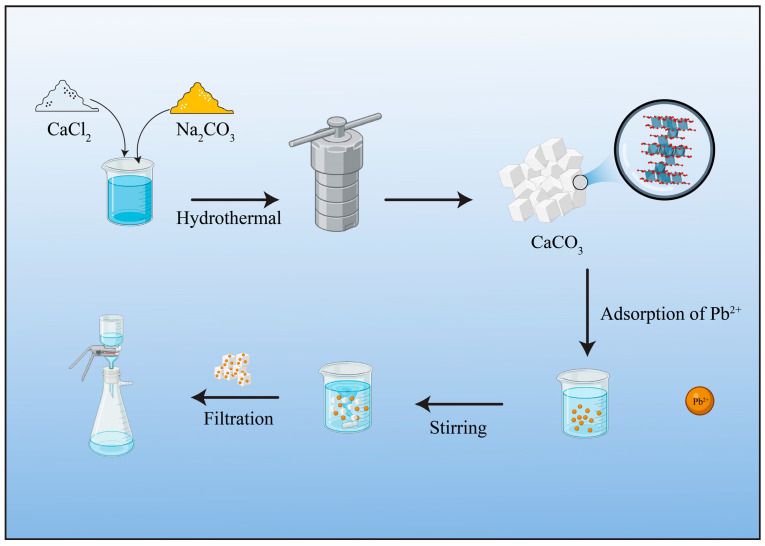
Schematic illustration of the fabrication of CaCO_3_ microcubes/parallelepipeds and adsorption of Pb(II).

Herein, CaCO_3_ was synthesized through chemical precipitation followed by a hydrothermal process and used as an adsorbent to remove Pb(II) from an aqueous solution (Figure 1). The crystal structure, microstructures, and chemical composition were analyzed by various characterization techniques. FTIR analysis revealed that the main carbonate peak of CaCO_3_ shifts towards a lower wavenumber (1386 cm^−1^) compared with the reported literature (1400–1500 cm^−1^), indicating changes in surface chemistry that may enhance the Pb(II) removal efficiency [21,28]. The adsorbent achieved a maximum uptake capacity of 4018 mg/g in a 100 ppm Pb(II) aqueous solution at a low dose of 0.01 g/L. The Freundlich isotherm model estimated an adsorption capacity of 1989.78 mg/g. The fitted adsorption data indicate that the pseudo-second-order model can delineate the adsorption kinetics. Post-adsorption analysis indicated that the primary removal mechanism was ion exchange between Ca^2+^ and Pb(II) on the surface of CaCO_3_.

## 2. Materials and Methods

### 2.1. Materials

All chemicals (Pb(NO_3_)_2_, Na_2_CO_3_, CaCl_2_) used in this study were analytical-grade and used as received without further purification. All solutions were prepared using ultrapure deionized (DI) water from the Millipore Simplicity 185 system. NaOH and HCl were used to adjust the pH values of solutions.

### 2.2. Preparation of CaCO_3_ Microcubes/Parallelepipeds

In a typical synthesis procedure, solutions A and B were prepared at room temperature by dissolving Na_2_CO_3_ (1.06 g) and CaCl_2_ (1.51 g) into 10 mL DI H_2_O using different flasks and then mixing both solutions. Subsequently, the solution was transferred into a 100 mL Teflon-lined autoclave for the hydrothermal reaction at 100 °C for 6 h. Finally, the product was collected, washed with DI H_2_O, and dried overnight at 70 °C.

### 2.3. Characterizations

The crystal structures were obtained using X-ray diffraction (XRD, Brooke, Germany KG223634) with Cu K_α_ radiation at a scan rate of 5° min^−1^. Fourier Transform Infrared (FTIR) spectra of the materials were recorded at a 400–4000 cm^−1^ wavenumber range using a Thermo-Fisher KG221452 (USA) Fourier Transform Infrared Detector. The microstructure and surface morphology were analyzed by scanning electron microscopy (SEM, Thermo-Fisher KG222748, Scios2 Hivac, USA) and transmission electron microscopy (TEM, Nanotemper KG220501, Prometheus NT. Plex, USA). The surface composition and the valences of the elements in materials were examined using X-ray photoelectron spectroscopy (XPS, Thermo Fisher ESCALAB 250Xi, USA). The Raman spectrum of CaCO_3_ was obtained using a high-resolution multifunction Raman spectrometer (KG230407, Japan) with laser light of a 512 nm wavelength. The specific surface area of CaCO_3_ was calculated by the multipoint Brunauer–Emmett–Teller (BET) method using adsorption data, and the pore size distributions were determined from the adsorption branch of the nitrogen isotherms by the Barret–Joyner–Halenda (BJH) model.

### 2.4. Pb(II) Uptake Experiment

The Pb(II) removal by CaCO_3_ microcubes/parallelepipeds was examined via batch experiments at room temperature. Typically, a Pb(II) solution (100.0 mL) with a 100.0 mg/L concentration and pH 6.0 was poured into a 250 mL canonical flask. The CaCO_3_ microcubes/parallelepipeds were added to this flask and then stirred gently for a given time. We used a 300 rmp stirring rate during the adsorption process because optimized stirring can inhibit particle aggregation and enhance the diffusion, contact efficiency, and mass transfer. To study the effect of CaCO_3_ dosage on the extraction of Pb^2+^, 1, 2, 4, 6, 8, and 10 mg of CaCO_3_ microcubes/parallelepipeds was added into 5 conical flasks each containing 100 mL of 100 mg/L Pb(II) solution with an initial solution pH of 6.0. The influence of the pH on the Pb(II) removal efficiency was inspected by stirring 10 g of adsorbent (CaCO_3_) and a series of 100 mL Pb(II) solutions (100 mg/L concentration) with changing pH values ranging from 3 to 11. After magnetic stirring at 300 rpm for a specific time, the supernatants were filtered through filter paper (0.45 μm), and exact residual Pb(II) concentrations were analyzed employing an inductively coupled plasma atomic emission spectrometer (ICP-AES, Agilent KG222164, Malaysia).

### 2.5. Data Analysis

The removal efficiency (%) and Pb(II) adsorption capacity of the examined microcubes/parallelepipeds at a given time (q_t_) were determined by Equations (1) and (2), respectively.

(1) $$\mathrm{Removal efficiency}=\frac{C_{0}{\;-\;C}_{t}}{C_{0}}\times 100\%$$

(2) $$q_{t}=\frac{C_{0}{-C}_{t}}{m}\;V$$

where C_0_, C_t_, and C_e_ are the adsorbate (Pb(II)) concentrations (mg/L) at the initial stage, time t, and equilibrium, respectively. V (L) is the volume of the solution, m (g) is the weight of the adsorbent, and q_t_ (mg/g) is the adsorption capacity of the adsorbent at time t.

The pseudo-first-order and pseudo-second-order kinetic models were used to study the adsorption kinetic mechanism of CaCO_3_ microcubes/parallelepipeds as presented using the following equations:

log(q_e_ − q_t_) = log(q_e_) − k_1_t (3)

(4) $$\frac{t}{q_{t}}=\frac{1}{k_{2}.q_{t}^{2}}+\frac{t}{q_{e}}$$

where q_e_ (mg/g) is the adsorption capacity of the adsorbent at equilibrium, and q_t_ (mg/g) is the adsorption capacity (mg/g) of the adsorbent at time t. k_1_ (min^−1^) is the equilibrium rate constant in the pseudo-first-order kinetic model, and k_2_ (g/mg·min) is the equilibrium rate constant in the pseudo-second-order kinetic model.

The Langmuir, Freundlich, and Temkin adsorption isotherms were used to illustrate adsorption data at different dosages of adsorbent. The Langmuir adsorption model can be expressed as shown in Equation (5).

(5) $$\frac{1}{q_{e}}=\frac{1}{q_{\max}}+\frac{1}{{{K_{L}q}_{\max}C}_{e}}$$

where q_e_ (mg/g) and C_e_ (mg/L) are the adsorption capacity and adsorbate concentration at equilibrium, respectively. q_max_ (mg/g) is the maximum monolayer adsorption capacity, and K_L_ shows the affinity between the adsorbent and adsorbate.

The Freundlich model has the following expression:

(6) $${\mathrm{lnq}}_{e}={\mathrm{lnk}}_{F}+\frac{1}{\;n}{\mathrm{lnC}}_{e}$$

where K_F_ and n are the adsorption capacity and adsorption intensity, respectively. The favorability of adsorption can be categorized based on n values as follows: n ˂ 1 (poor), 1 ˂ n ˂ 2 (moderately difficult), and 2 ˂ n ˂ 10 (favorable adsorption) [29].

The Temkin isotherm model gives adsorbent–adsorbate interactions and is presented by Equation (7).

(7) $$q_{e}=\frac{RT}{b_{T}}\ln\left(A_{T}\right)+\frac{\mathrm{RT}}{b_{T}}\ln{(C}_{e})$$

where b_T_ (J/mol) is the Temkin isotherm constant related to the heat of sorption, A_T_ (L/g) is the Temkin isotherm equilibrium binding constant, T (298 K) is the absolute temperature, and R (8.3145 J/mol/K) is the universal gas constant.

## 3. Results and Discussion

### 3.1. Morphological and Structural Characterizations of CaCO_3_

The CaCO_3_ microcubes/parallelepipeds were synthesized using simple precipitation followed by a hydrothermal method (Figure 1). The precipitation reaction occurred during the mixing of Na_2_CO_3_ and CaCl_2_ according to the following equation:

Na_2_CO_3(aq)_ + CaCl_2(aq)_ + H_2_O → CaCO_3(s)_ + 2NaCl_(aq)_ + H_2_O (8)

**Figure 2:**
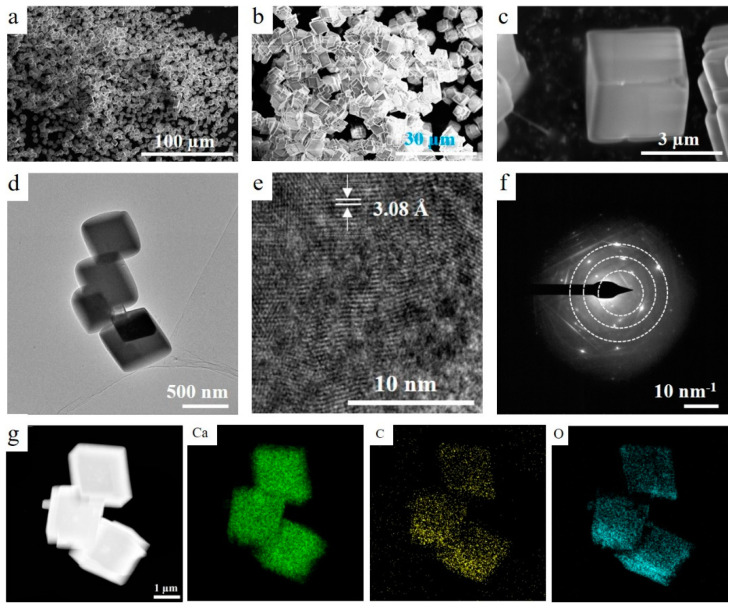
Surface morphology of CaCO_3_ microcubes/parallelepipeds: (**a**–**c**) SEM, (**d**) TEM, (**e**) HRTEM, and (**f**) SAED pattern of CaCO_3_ microcubes/parallelepipeds and (**g**) HAADF-STEM and corresponding element mapping of CaCO_3_.

The morphological and structural properties of the as-synthesized CaCO_3_ were observed using SEM and TEM. All the particles show a microcube/parallelepiped-like morphology with smooth faces and well-defined edges, as shown in Figure 2a–c. The TEM micrographs further revealed that CaCO_3_ is composed of microcubes/parallelepipeds with an edge length of 0.1–3.0 µm and a relatively smooth surface (Figure 2d). HRTEM (high-resolution TEM) displayed that the interlayer distance of CaCO_3_ is ≈3.08 Å, matching the (104) plane (Figure 2e). The corresponding SAED (selected-area electron diffraction) pattern of CaCO_3_ is provided in Figure 2f and is in accordance with the XRD pattern. Figure 2g and Figure S1 (Supporting Information) illustrate the HAADF-STEM (high-angle annular dark-field scanning TEM) and corresponding EDX pattern and elemental mapping of CaCO_3_ microcubes/parallelepipeds. The EDX pattern reveals a set of signals corresponding to the Ca, C, and O elements, and all these elements (Ca, C, and O) are uniformly and densely distributed within the entire microcube, as shown by their elemental mapping images.

**Figure 3:**
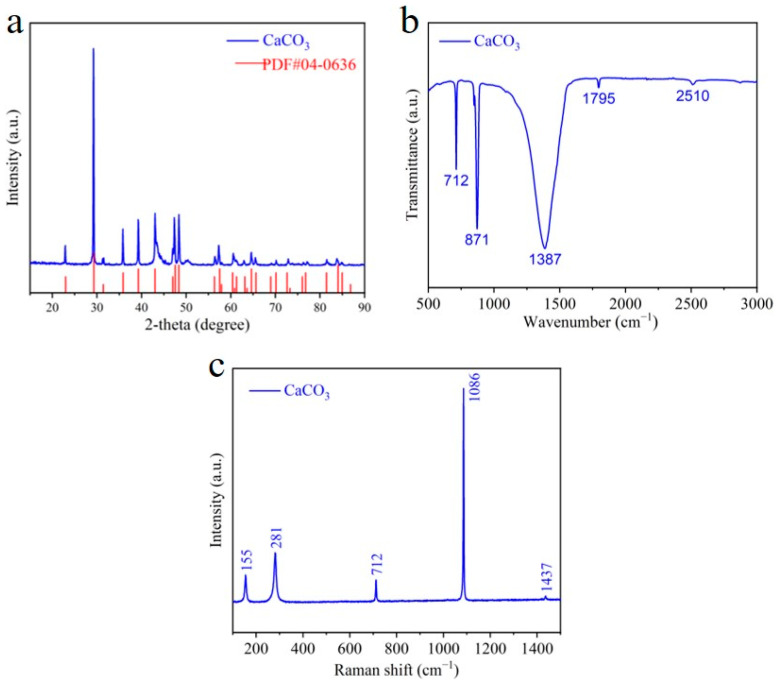
Structural characterization of CaCO_3_ microcubes/parallelepipeds: (**a**) XRD pattern, (**b**) FTIR analysis, and (**c**) Raman spectra of CaCO_3_.

The XRD pattern in Figure 3a presents a deep understanding of the crystal structure of CaCO_3_ microcubes/parallelepipeds. All the distinct peaks agree with the hexagonal calcite phase of CaCO_3_ (PDF#04-0637), confirming the single-crystal-phase structure of microcubes/parallelepipeds with high purity. This calcite phase is the most stable phase of CaCO_3_ [21]. Moreover, the sharp peaks in the XRD pattern specify the highly crystalline nature of CaCO_3_, which plays a critical role in enhancing the adsorption capacity and stability of the material. Specifically, the prominent diffraction peaks at 22.9°, 29.2°, 35.8°, 39.2°, 43.0°, 47.3°, 48.3°, 56.4°, 57.3°, 60.6°, 64.6°, 65.5°, 70.2°, 72.9°, and 81.6° are attributed to the (012), (104), (110), (113), (202), (024), (116), (211), (122), (214), (300), (0012), (0210), (128), and (2110) crystallographic planes of calcite, respectively. This stable and highly crystalline phase may play an important role in evaluating the adsorption capacities and stability of the material.

The chemical structure of CaCO_3_ was determined by the FTIR spectrometer. The FTIR spectrum shows three prominent adsorption peaks at 712, 871, and 1386 and two minor peaks at 1795 and 2510 cm^−1^ (Figure 3b). The prominent adsorption peaks at 712, 871, and 1386 cm^−1^ can be assigned to carbonate in-plane bending (*v*_4_), carbonate out-of-plane bending (*v*_2_), and the asymmetric stretching vibration of carbonate (*v*_3_) of the calcite phase, respectively [4,30,31]. In our case, the carbonate peak at 1400–1500 cm^−1^ shifted towards the lower wavenumber (1386 cm^−1^) compared with reported values, indicating a change in the chemical bonding or environment of carbonate ions in the CaCO_3_ molecule [4]. This shift can be attributed to a decrease in the bond strength or an increase in the bond length of carbonate ions during the precipitation reaction, which may enhance the Pb adsorption. The XRD and FTIR analysis was further corroborated by Raman spectra. Figure 3c depicts the Raman spectra of calcite CaCO_3_ microcubes/parallelepipeds. The most intense peak at 1086 cm^−1^ can be assigned to the A_1g_ internal mode resulting from the v_1_ symmetric stretching vibration of Ca_3_^2−^. The v_4_ in-plane bending vibration of the carbonate group is detected at 712 cm^−1^ [32]. The peaks at 281 cm^−1^ and 155 cm^−1^ are attributed to the translational and rotational vibrations of the CaCO_3_ lattice [32].

**Figure 4:**
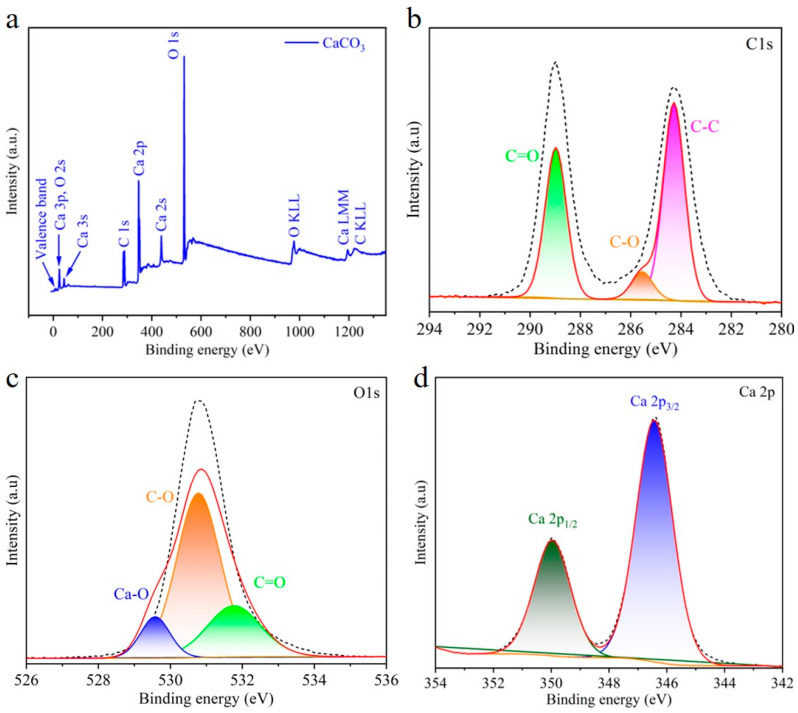
Surface chemistry and chemical composition of CaCO_3_: (**a**) Survey spectrum of CaCO_3_. High-resolution spectra of (**b**) C 1s, (**c**) O 1s, and (**d**) Ca 2P in CaCO_3_.

XPS was used to analyze the chemical composition and valence state of elements in the CaCO_3_ microcubes/parallelepipeds. The XPS survey spectrum of CaCO_3_ microcubes/parallelepipeds revealed photoelectrons and Auger electron signals (induced by X-ray) of Ca, C, and O_2_, which further confirm the high purity and chemical composition of the CaCO_3_ microcubes/parallelepipeds (Figure 4a). The high-resolution XPS spectra of individual elements are presented in Figure 4b–d. In the high-resolution Ca 2p spectrum, the two peaks located at 346.4 and 350.1 eV were assigned to Ca 2p_3/2_ and Ca 2p_1/2_, respectively, indicating a 2+ valence state for Ca (Figure 4b) [33]. The C 1s spectrum can be deconvoluted into three components located at 288.97, 285.43, and 284.28 eV, which can be ascribed to C=O, C-O, and C-C, respectively (Figure 4c) [19]. Further, the O 1s high-resolution spectrum showed three fitted peaks at 529.56, 530.78, and 531.8 eV, corresponding to Ca-O, C-O, and C=O bonds, respectively (Figure 4d). Moreover, the N_2_ adsorption–desorption isotherm of CaCO_3_ and the corresponding pore size distribution curve are presented in Figure S2, Supplementary Information. The Brunauer–Emmett–Teller (BET) surface area of CaCO_3_ was calculated to be 5.7 m^2^ g^−1^. The BET surface area of pure CaCO_3_ is relatively low compared to modified materials because its highly crystalline and crystal structure is tightly packed and highly ordered [1,21]. The surface of the crystal is flat and smooth, with minimal surface roughness and impurity. Consequently, there are relatively few active sites available, and the total surface area is low.

### 3.2. Adsorption Properties of Pb(II) on CaCO_3_ Microcubes/Parallelepipeds

#### 3.2.1. Effect of pH, Adsorption Period, and Adsorbent Dosage

**Figure 5:**
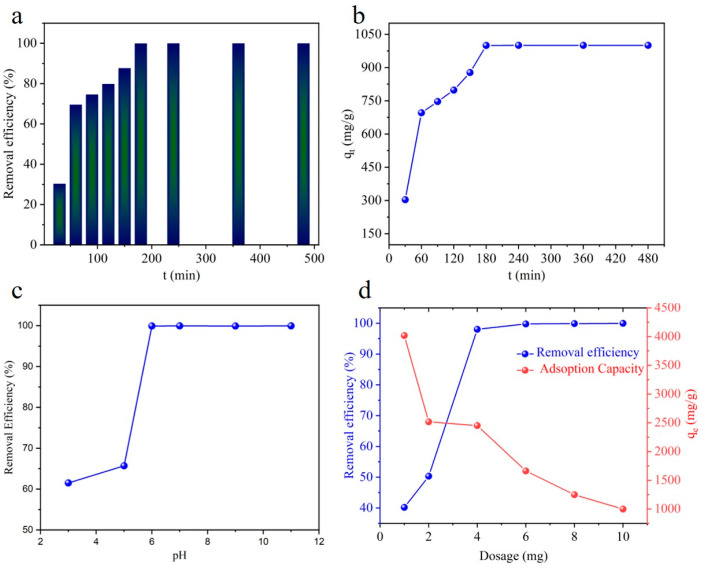
Effect of adsorption period on the (**a**) removal efficiency and (**b**) capacity of Pb(II): C_0_ = 100 mg/L, adsorbent dose = 10 mg, and pH = 6.0. (**b**,**c**) Effect of pH on adsorption of Pb(II): C_0_ = 100 mg/L, adsorbent dose = 10 mg, and adsorption period 180 min. (**d**) Effect of adsorbent dose on the adsorption of Pb(II): C_0_ = 100 mg/L, adsorption period = 180 min, and pH = 6.

To evaluate the removal effects of calcite CaCO_3_ microcubes/parallelepipeds on Pb(II) in aqueous solutions, a batch of experiments was conducted, and ICP-MS was used to calculate Pb(II) ion concentrations. The measurements of the adsorption period describe the quickness of the extraction of heavy metal ions by the adsorbent materials and the optimal time for the extraction of heavy metal ions. To optimize the adsorption period, the adsorption of Pb onto calcite CaCO_3_ microcubes/parallelepipeds at room temperature, C_0_ = 100 mg/L, adsorbent dosage = 10 mg, and pH = 6 was conducted. Figure 5a reveals that the removal efficiency increased quickly with increasing the adsorption period and then reached equilibrium. The adsorption is quick due to a fast ion-exchange reaction between Pb(II) and CaCO_3_, which will be explained in the next section. It takes 180 min to achieve a 99.99 % removal efficiency of Pb(II) from a 100 mg/L aqueous solution. Figure 5b demonstrates the microcubes/parallelepipeds’ adsorption capacity (q_t_) as a reaction time function. The q_t_ for Pb(II) increased intensely within 60 min (with q_t_ = 695.5 mg/g), and then the adsorption rate became slower with the prolonged adsorption period until reaching equilibrium within 180 min with q_e_ of 999.9 mg/g, and the capacity remained stable after 180 min. A rapid increase in the q_t_ for adsorbing Pb(II) represents a decent adsorption capability to remove Pb(II). The optimized adsorption period for Pb(II) onto CaCO_3_ was considered to be 180 min for all further experiments.

The adsorption behavior of calcite CaCO_3_ was also evaluated with the effect of pH, as the pH value is a vital monitoring parameter during the adsorption process. The initial concentration of Pb(II) ions was 100 mg/L at room temperature. The pH values were changed from 3 to 11. The results show that the removal efficiency reaches up to 61.5% within 180 min at pH 3, which then increases with pH in acidic conditions and reaches the maximum (~99.9%) at pH 6.0 (Figure 5c). The low adsorption of Pb(II) at pH < 6 can be ascribed to the partial dissolution (i.e., incapability of CaCO_3_ to exist stably) under acidic conditions [2,22,34]. With further increases in the pH values, the removal efficiency did not change significantly and almost remained at the maximum (99.9%) in the pH range from 6 to 11. It is well known that the adsorbent surface charge has a significant effect on the removal of anions due to Coulombic attraction [35]. Reportedly, the surface charge on CaCO_3_ is positive in the pH range of 5–10 [24]. However, Pb(II) is a cation, and the maximum removal efficiency (99.9%) was attained at a pH range of 6~11, implying that the adsorbent surface charge did not affect the Pb(II) removal in our system. Therefore, Pb(II) adsorption onto CaCO_3_ can be attributed to the ion-exchange phenomenon between Pb(II) (cations) and Ca^2+^ (cations) on the adsorbent surfaces [24].

The adsorbent dosage is one of the most critical parameters for studying the adsorption behavior. The influence of the dosage on the extraction of Pb(II) with an adsorption period = 180 min, pH = 6, and C_0_ = 100 mg/L was evaluated by varying the dosage from 1.0 to 10.0 mg. The absorptivity of Pb(II) increased with an increase in the dosage concentration due to the availability of a large number of active sites for the ion-exchange phenomenon. The saturation point for Pb(II) was observed at a dosage of 0.04 g/L (4 mg) for CaCO_3_ with a 98.04% removal efficiency and an adsorption capacity of 2451 mg/g (Figure 5d). The maximum adsorption capacity of 4018 mg/g was attained with a 40.18% removal efficiency at a dosage of 0.01 g/L (Figure 5d). Table 1 highlights the comparative study of CaCO_3_-based adsorbents for the removal of Pb(II). The adsorption capacity for Pb(II) obtained in our study exceeds that of previously reported CaCO_3_-based materials, indicating that it is an efficient adsorbent for Pb(II) remediation.

#### 3.2.2. Adsorption Kinetics and Isotherm Studies of Pb(II) Removal by CaCO_3_

To know the adsorption kinetic mechanism of CaCO_3_, pseudo-first-order and pseudo-second-order equations are applied to understand adsorption dynamics. We applied pseudo-kinetic models because they are often derived from empirical discussion, are simple in calculation, and have been extensively used in the literature [16,17,18,19,20,21,22,23]. These models are employed to streamline the analysis of complex adsorption processes, well suited to limited datasets, and facilitate straightforward calculations, enabling the effective assessment of initial adsorption rates. Additionally, they support comparative analysis with prior studies, contributing to the broader validation of results. We first fitted the pseudo-first- and pseudo-second-order models for the first four points. The R^2^ value of the pseudo-first-order model (0.8701) is higher than that of the pseudo-second-order model (0.7539) (Figure S3a,b, Supporting Information), indicating a dominant physisorption process in the initial process of adsorption due to the availability of abundant active sites. However, with the passage of time, the dominant chemisorption process was observed in the adsorption process (Figure S3c,d, Supporting Information).

**Figure 6:**
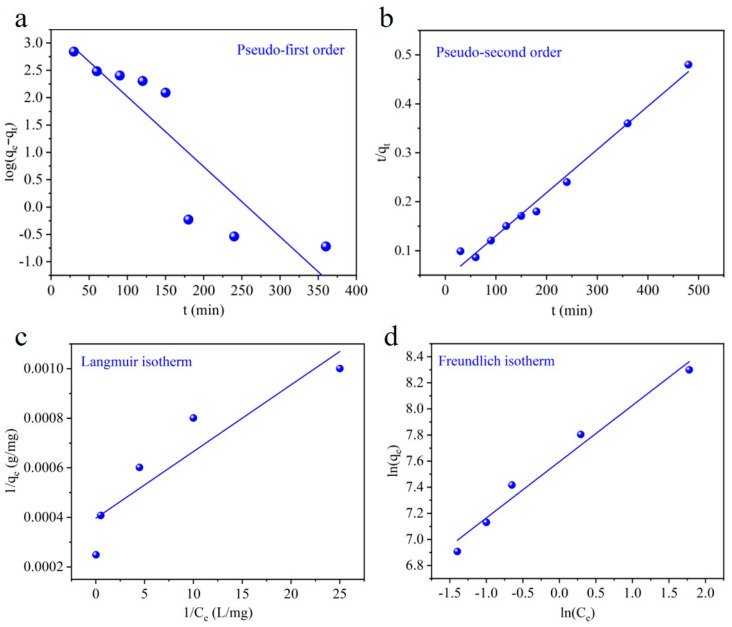
(**a**) Adsorption kinetics modeled with the pseudo-first-order equation and (**b**) pseudo-second-order equation**.** Adsorption isotherms of Pb(II) adsorbed onto CaCO_3_ fitted by the (**c**) Langmuir model and (**d**) Freundlich model.

The adsorption kinetic plots for the whole adsorption process obtained after fitting these two models are depicted in Figure 6a,b, and the related kinetic parameters are listed in Table 2. The pseudo-second-order model gained a linear regression coefficient of over 0.9866, which is much higher than that of the pseudo-first-order model (0.7991). This demonstrates that the pseudo-second-order model is predominant in defining the kinetic mechanism for this adsorbent system. Moreover, the theoretical capacity (q_e_ = 1130 mg/g) calculated by the pseudo-second-order model is closer to the experimental data. This indicates that the overall adsorption process is mainly controlled by the chemisorption of Pb(II) onto CaCO_3_ instead of physisorption. Thus, the nature of the removal of Pb(II) with CaCO_3_ could be simulated with a pseudo-second-order model.

To further study the interaction between CaCO_3_ and Pb(II) at equilibrium, the adsorption isotherms were investigated using a 100 mg/L concentration of Pb(II) with various dosages (1–10 mg) at equilibrium (i.e., for 180 min) and pH 6.0. The Langmuir, Freundlich, and Temkin isotherm models are applied in this study, and corresponding fitting curves are depicted in Figure 6c,d and Figure S4. Table 3 provides the parameters obtained from the linear fitting of the Langmuir, Freundlich, and Temkin isotherm models. The results revealed that the adsorption data of Pb(II) fit well with the Freundlich isotherm model compared with the Langmuir model, indicating the multilayer adsorption of Pb(II) onto CaCO_3_.The adsorption capacity computed by the Freundlich equation was 1989.78 mg/g. Moreover, the Freundlich model parameter n, which is roughly an indicator of the adsorption intensity, was 2.3192, showing the favorable adsorption condition of Pb(II) onto CaCO_3_ and that mainly the chemisorption mechanism was involved during the adsorption process. The Temkin model further showed moderate to strong intermolecular interactions between Pb(II) and the CaCO_3_ adsorbent. Moreover, the higher value of A_T_ (10.61 L/g) in the Temkin model further showed relatively abundant active sites available on the adsorbent surface for adsorption. Moreover, the low value of b_T_ (2.5853) further proposed that both physisorption and chemisorption may be involved in the CaCO_3_ adsorption process.

#### 3.2.3. Mechanism of Pb(II) Removal by Calcite CaCO_3_ Microcubes/Parallelepipeds

**Figure 7:**
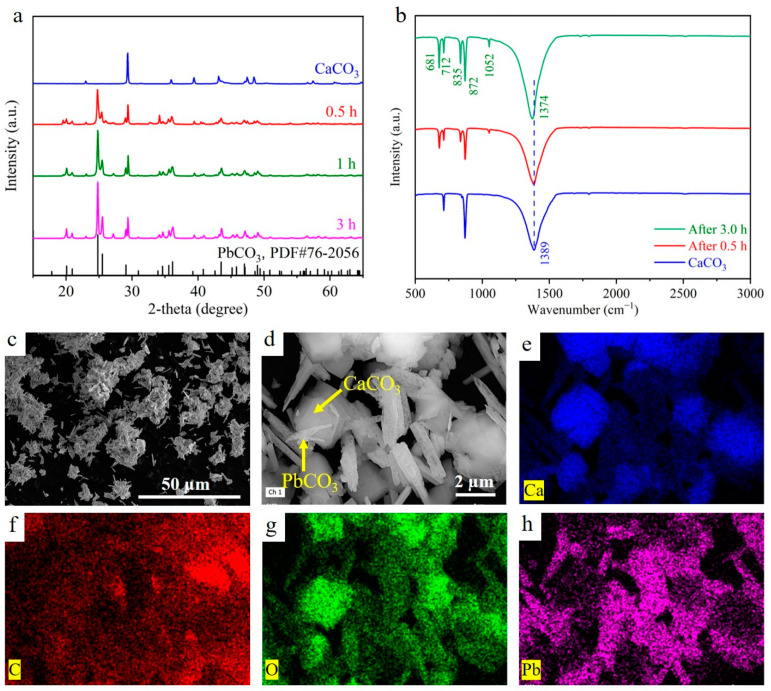
(**a**) The crystal phase evolution of CaCO_3_ during Pb(II) removal. (**b**) FTIR spectrum of CaCO_3_ before and after Pb(II) adsorption. (**c**,**d**) SEM and (**e**–**h**) corresponding elemental mapping of Pb(II) adsorbate CaCO_3_.

To corroborate the adsorption mechanism of CaCO_3_, XRD, FTIR, SEM, EDX, and XPS tests were performed to examine the morphologies and structural and chemical compositions of CaCO_3_ after the adsorption of Pb(II). The crystal phase evolution during Pb(II) extraction is presented in Figure 7a. The adsorbed Pb(II) samples were obtained after different durations of reactions (0.5, 1.0, and 3.0 h). After 0.5 h of contact time, discrete peaks aligned with the cerussite (PbCO_3_), indicating the fast formation of PbCO_3_ or the rapid removal of Pb(II) by CaCO_3_. Concurrently, a reduction in the peak intensities of calcite was also found. Notably, the cerussite phase increased and the calcite phase decreased with the increase in the reaction time. At the equilibrium stage (3.0 h), the maximum intensity of the cerussite phase is found in the XRD pattern, suggesting that most of the CaCO_3_ adsorbent transformed into PbCO_3_ via the ion-exchange reaction after Pb(II) removal.

The FTIR spectrum of the CaCO_3_ microcubes/parallelepipeds before and after adsorbing Pb(II) is shown in Figure 7b. The spectra of CaCO_3_ before adsorption reveal three prominent vibrational peaks of calcite at 712, 872, and 1386 cm^−1^. However, three new peaks near 681, 835, and 1050 cm^−1^ (assigned to cerussite) after taking up Pb(II) were observed, confirming the formation of cerussite (PbCO_3_) and that Pb(II) was adsorbed through ion exchange. Interestingly, after 0.5 h, the intensity of the carbonate bands at 712 and 871 cm^−1^ decreased, while the peak intensity at 1386 cm^−1^ remained almost unchanged. These results show that these carbonate groups are involved in ion-exchange reactions with Pb(II). In contrast, after 3.0 h, these peak intensities did change; the carbonate C-O vibration peak intensity increased and shifted from 1387 cm^−1^ to a low-wavenumber region of 1374 cm^−1^ after adsorption, indicating that Pb was ion-exchanged with Ca^2+^ instead of CO_3_^2−^. The red shift of 12 cm^−1^ may be attributed to the decreasing mass of CaCO_3_ and the repulsive forces between the adsorbent and Pb(II). From the FTIR analysis, it can be concluded that the ion-exchange reaction was involved due to carbonate groups at the beginning of the adsorption process. However, at the equilibrium stage of adsorption, Pb was ion-exchanged with Ca(II).

To further confirm this, the adsorbed Pb(II) sample was examined using SEM equipped with EDS. Tabular and needle-like crystals were formed and agglomerated on the calcite surfaces (Figure 7c,d). The residual CaCO_3_ microcubes/parallelepipeds still retained the original morphology with a homogenous distribution of Ca, C, O, and Pb (Figure 7e,h and Figure S5, Supporting Information), revealing that the adsorption of Pb(II) does not have a significant effect on the microstructure of CaCO_3_. EDX further showed that the proportion of Ca decreased from 29.71 % to 17.36 % by weight after adsorbing Pb (Figures S1 and S6, Supporting Information), showing an ion-exchange reaction. The residual CaCO_3_ may serve as a pH buffer in the solution, enabling the efficient removal of Pb(II) by the adsorbent with a broad pH range.

**Figure 8:**
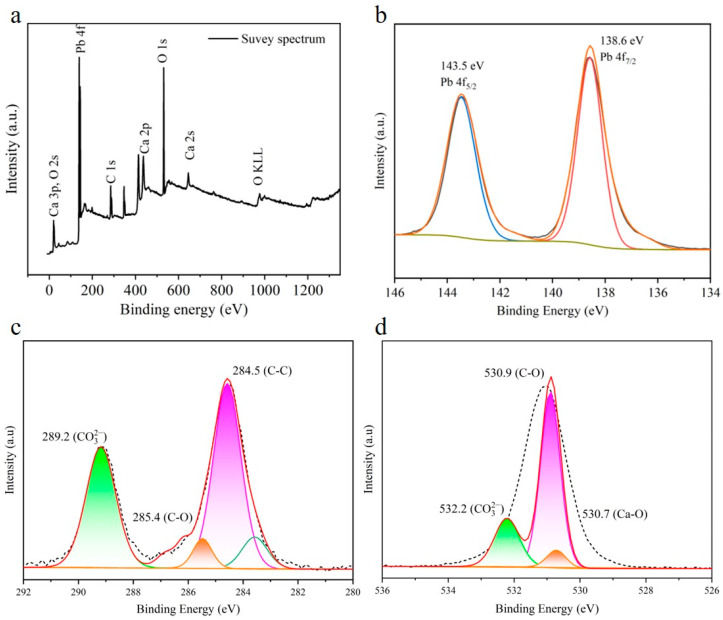
(**a**) Survey spectrum of CaCO_3_ after Pb(II) adsorption. High-resolution spectra of (**b**) Pb 4f, (**c**) C 1s, and (**d**) O 1s of CaCO_3_ after Pb(II) adsorption.

Figure 8a reveals the XPS survey of the adsorbed Pb(II) sample which shows three peaks for C 1s, O 1s, and Ca 2p and one additional peak for Pb 4f, confirming the adsorption of Pb. The Pb adsorption mechanism was elucidated by high-resolution spectra of O 1s, C 1s, and Pb 4f. In the high-resolution Pb 4f spectrum, two obvious peaks located at 138.58 and 143.48 eV were assigned to Pb 4f_7/2_ and Pb 4f_5/2_, respectively, indicating the +2 valence state of Pb in the recovered sample (Figure 8b). Two broad and weak peaks at 137.68 and 142.68 eV were also found in the high-resolution spectrum of Pb, which are attributed to the oxidation of Pb or the presence of Pb(OH)_2_ [4,45,46]. This indicates that a slight amount of Pb(OH)_2_ might be present in the treated sample, which was not detected in the XRD pattern due to its small content. The high-resolution XPS spectra of C can be split into three peaks assigned to C-C, C-O, and CO_3_^2−^, while the deconvoluted O1s spectra showed three peaks for Ca-O, C-O, and CO_3_^−2^ (Figure 8c,d). Further, to elaborate on the adsorption mechanism, we measured the difference between the binding energy of CO_3_^2−^ in the XPS spectra of CaCO_3_ microcubes/parallelepipeds before and after adsorption. The differences in the binding energy for C1s and O1s corresponding to CO_3_^2−^ were calculated to be 0.13 and 0.4 (Figure 4b,c and Figure 8c,d), respectively, suggesting the rearrangement of the active sites’ carbonate ions (CO_3_^2−^) after Pb(II) adsorption, bringing about easier ion exchange between Ca^2+^ and Pb(II). These findings further corroborate that an ion-exchange reaction happens during the adsorption of Pb by CaCO_3_.

## 4. Conclusions

Cost-effective CaCO_3_ microcubes/parallelepipeds were fabricated by a co-precipitation and hydrothermal process, characterized by various techniques, and used as an adsorbent for Pb(II) removal from an aqueous solution. The FTIR spectrum revealed the main carbonate peak at 1386 cm^−1^, lower than the reported values in the literature, suggesting a strong interaction between Pb(II) and the surface of CaCO_3_, leading to better Pb(II) removal efficiency. The CaCO_3_ revealed excellent removal activity for Pb(II), and the adsorption capacity reached 4018 mg/g. Meanwhile, a 99.99% removal efficiency was obtained within 180 min in a 100 ppm (100 mg/L) solution of Pb(II). Kinetic investigations showed that the chemisorption phenomenon happened in the removal of Pb(II). The Freundlich isotherm model described the adsorption behavior, and the uptaken capacity calculated by the Freundlich adsorption isotherm was 1989.78 mg/g. The Pb(II) adsorbed onto the surface of CaCO_3_ via ion-exchange reactions between Ca^2+^ and Pb(II). The excellent adsorption capacity, simple and green synthesis process, and low cost make CaCO_3_ an excellent candidate for treating wastewater polluted with heavy metals.

## Figures and Tables

**Table 1:** Comparison of adsorption capacities for Pb(II) of various CaCO_3_-based materials.

CaCO_3_-Based Adsorbent	HM	Adsorption Capacity (mg/g)	References
Mg-CaCO_3_ LSs	Pb(II)	1961.9	[2]
MoS_2_B/CaCO_3_/Alg	Pb(II)	833.3	[5]
Ca-MBHC	Pb(II)	475.58	[36]
HAP/BCC	Pb(II)	860	[37]
CaCO_3_/Fe_3_O_4_@ndcosane microcapsules	Pb(II)	497.6	[38]
CCS700	Pb(II)	299.98	[39]
MCCR-350-550	Pb(II)	1179–1350	[1]
nZVI@CaCO_3_	Pb(II)	3828	[40]
V-COS	Pb(II)	1884	[41]
Bio-CaCO_3_	Pb(II)	1667	[21]
TG/CC HNC	Pb(II)	192	[42]
IO@ CaCO_3_	Pb(II)	1041.9	[24]
n-CaCO_3_	Pb(II)	180.5	[43]
Chitosan/CaCO_3_	Pb(II)	98.03	[44]
CaCO_3_ microcubes/parallelepipeds	Pb(II)	4018	Current study

**Table 2:** The kinetic parameters for the pseudo-first-order and pseudo-second-order models. pH = 6.0, C_0_ = 100 mg/L, dosage = 10 mg, and T = 25 °C.

Pseudo-First Order			Pseudo-Second Order		
R^2^	K_1_ (min^−1^)	q_e_ (mg/g)	R^2^	K_2_ (min^−1^)	q_e_ (mg/g)
0.7991	0.0295	1992.508	0.9866	1.86 × 10^−5^	1130

**Table 3:** The isotherm parameters fitted by the Langmuir, Freundlich, and Temkin models of Pb(II) adsorption onto CaCO_3_: pH = 6.0, C_0_ = 100 mg/L, reaction time = 180 min, T = 25 °C, and dosage 1–10 mg.

Langmuir Constants			Freundlich Constants			Temkin Constants		
R^2^	K_L_ (L/mg)	q_m_ (mg/g)	R^2^	n	K_F_ (mg/g)	R^2^	b_T_ (J/mol)	A_T_ (L/g)
0.85541	14.7339	2521.76	0.97582	2.3192	1989.78	0.9965	2.5853	10.61

## Data Availability

The data that support the findings of this study are available on request from the corresponding author.

## Supplementary Materials

The following supporting information can be downloaded at: https://www.mdpi.com/article/10.3390/ma17225523/s1, Figure S1: EDX of CaCO_3_; Figure S2: (a) N_2_ adsorption/desorption isotherms of the CaCO_3_. (b) Pore-size distributions for the respective sample; Figure S3: Adsorption kinetics modeled with the pseudo-first order equation and pseudo-second order equation at different stages; Figure S4: Temkin isotherm model applied to adsorption of Pb(II) by CaCO_3_; Figure S5: HAADF image and corresponding elemental mapping of Pb(II) adsorbate CaCO_3_; Figure S6: EDX of CaCO_3_ after Pb(II) adsorption; Figure S7: High-resolution spectrum of Ca 2P_3/2_ after Pb(II) adsorption.
