# Supplementary material for: Fabrication of CaCO3 Microcubes and Mechanistic Study for Efficient Removal of Pb from Aqueous Solution
Source: Materials (Basel). 2024 Nov 12;17(22):5523. doi: 10.3390/ma17225523 (PMC11595343; doi:10.3390/ma17225523)
Supplement: Supplementary file 1 [file materials-17-05523-s001.zip › materials-3284374-supplementary.pdf]

Supporting Information

Fabrication of $CaCO_3$ Microcubes and Mechanistic Study for Efficient Removal of Pb from Aqueous Solution

Ufra Naseer [1,2], Asim Mushtaq [2], Muhammad Ali [2], Moazzam Ali [2], Atif Ahmad [3], Muhammad Yousaf [2,*] and Tianxiang Yue [1,*]

[1] State Key Laboratory of Resources and Environment Information System, Institute of Geographical Sciences & Natural Resources Research, Chinese Academy of Sciences, Beijing 100871, China
[2] ZJU-Hangzhou Global Scientific and Technological Innovation Center, Zhejiang University, Hangzhou 311200, China
[3] Department of Biological Geological and Environmental Science, University of Bologna, 40126 Bologna, Italy
* Correspondence: muhammadyousaf@zju.edu.cn (M.Y.); yuetx@igsnrr.ac.cn (T.Y.)

Contents

Figure S1: EDX of $CaCO_3$

Figure S2: (a) N_2 adsorption/desorption isotherms of the $CaCO_3$. (b) Pore-size distributions for the respective sample

Figure S3: Adsorption kinetics modeled with the pseudo-first order equation and pseudo-second order equation at different stages

Figure S4: Temkin isotherm model applied to adsorption of Pb(II) by $CaCO_3$

Figure S5: HAADF image and corresponding elemental mapping of Pb(II) adsorbate $CaCO_3$

Figure S6: EDX of $CaCO_3$ after Pb(II) adsorption

Figure S7: High-resolution spectrum of Ca $2P_{3/2}$ after Pb(II) adsorption

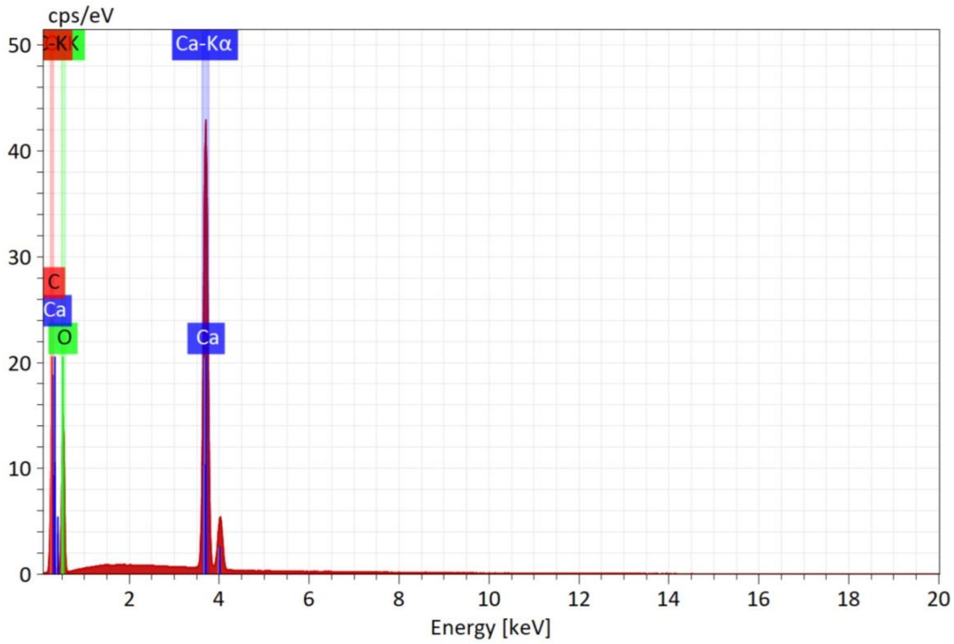

Element	Atomic Number	Weight Percentage (%)	Atom Percentage (%)
O	8	47.91	53.49
C	6	22.37	33.27
Ca	20	29.71	13.24
		**100.00**	**100.00**

Figure S1. EDX of $CaCO_3$.

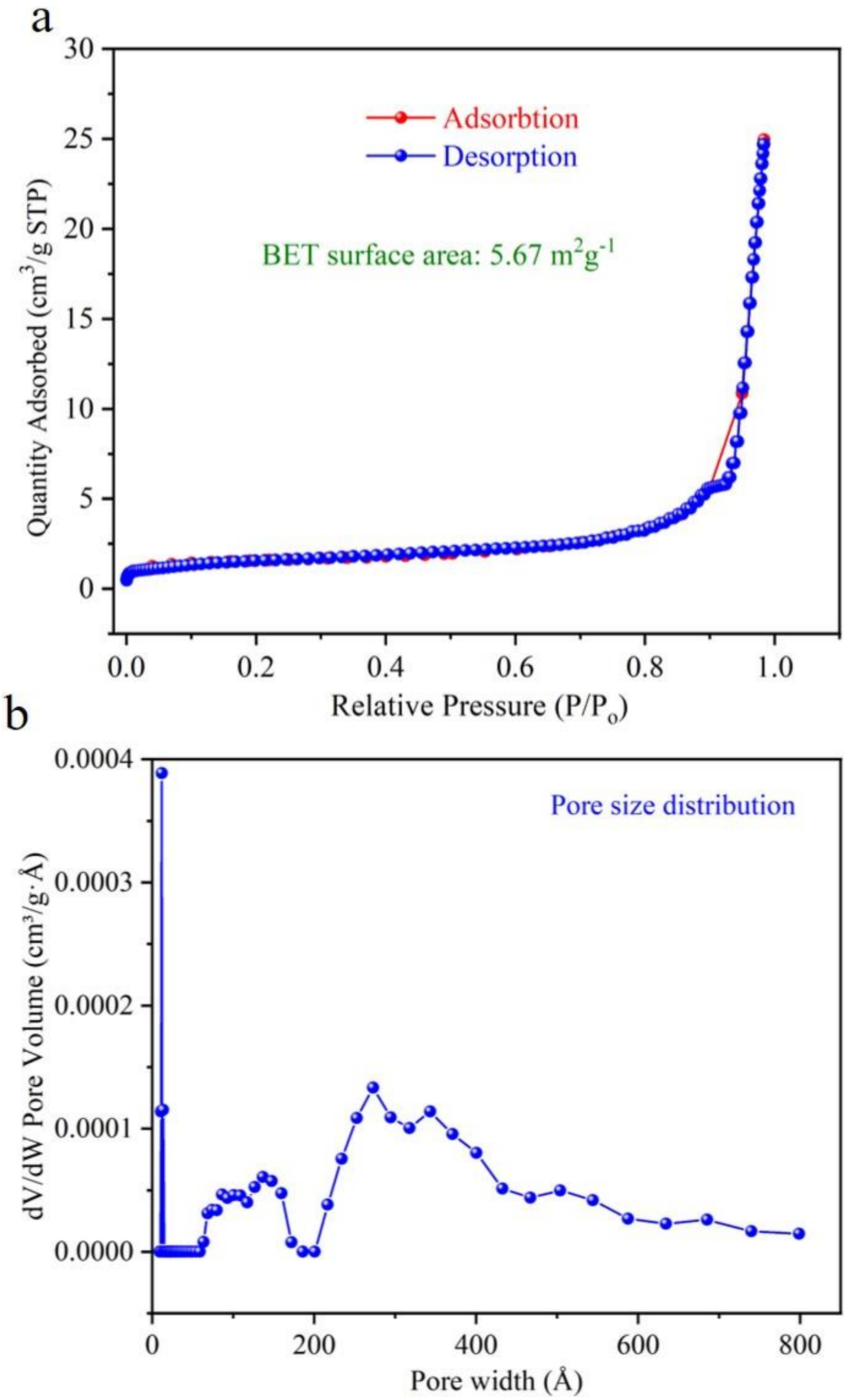

Figure S2. (a) N_2 adsorption/desorption isotherms of the $CaCO_3$. (b)Pore-size distributions for the respective sample.

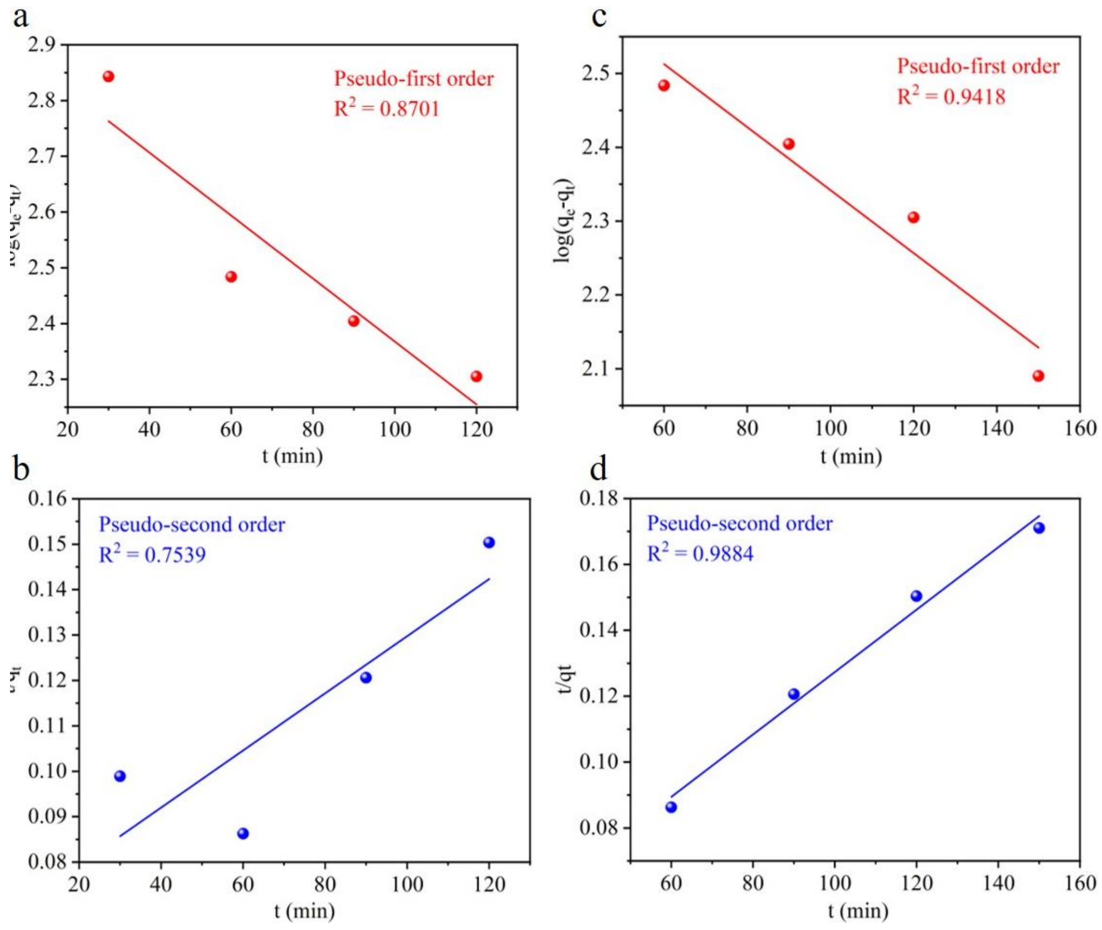

Figure S3. Adsorption kinetics modeled with the pseudo-first order equation and pseudo-second order equation at different stages.

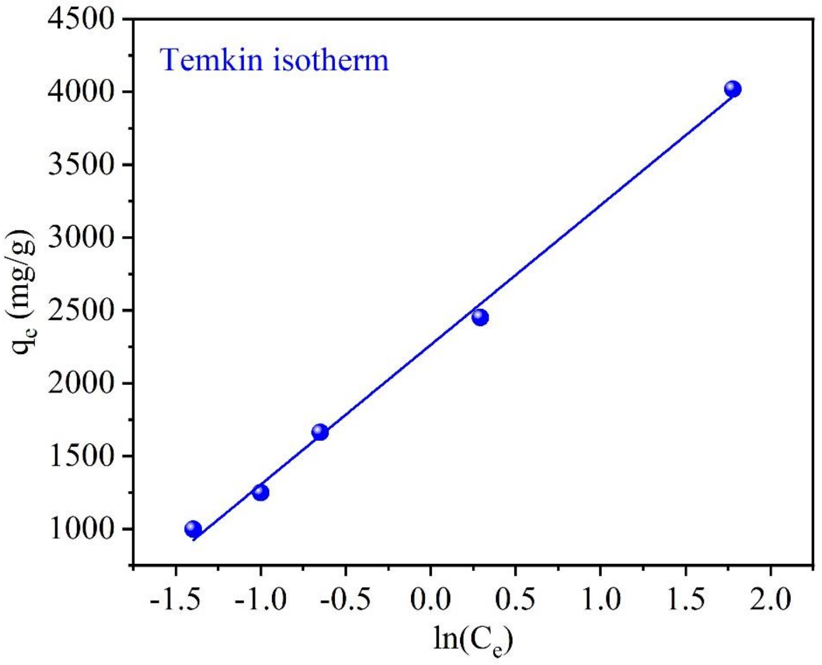

Figure S4. Temkin isotherm model applied to adsorption of Pb(II) by $CaCO_3$.

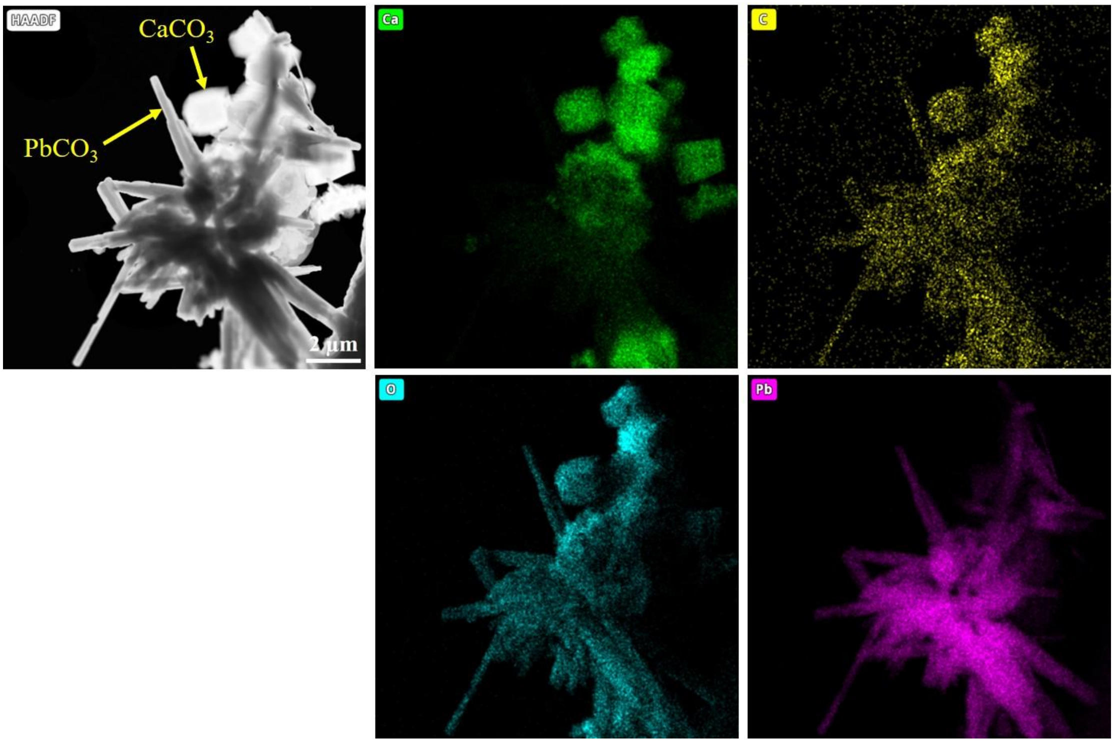

Figure S5. HAADF image and corresponding elemental mapping of Pb(II) adsorbate $CaCO_3$.

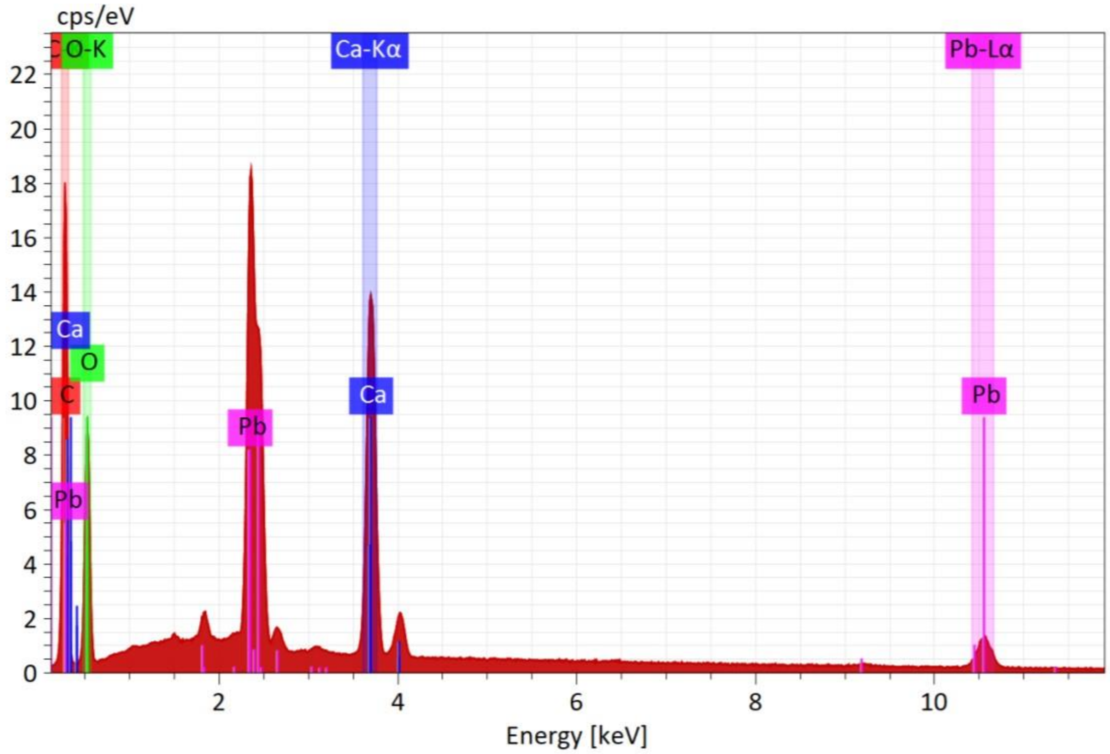

Element	Atomic Number	Weight Percentage (%)	Atom Percentage (%)
C	6	25.38	50.15
O	8	24.11	35.77
Ca	20	17.36	10.28
Pb	82	33.15	3.80
		**100.00**	**100.00**

Figure S6. EDX of $CaCO_3$ after Pb(II) adsorption.

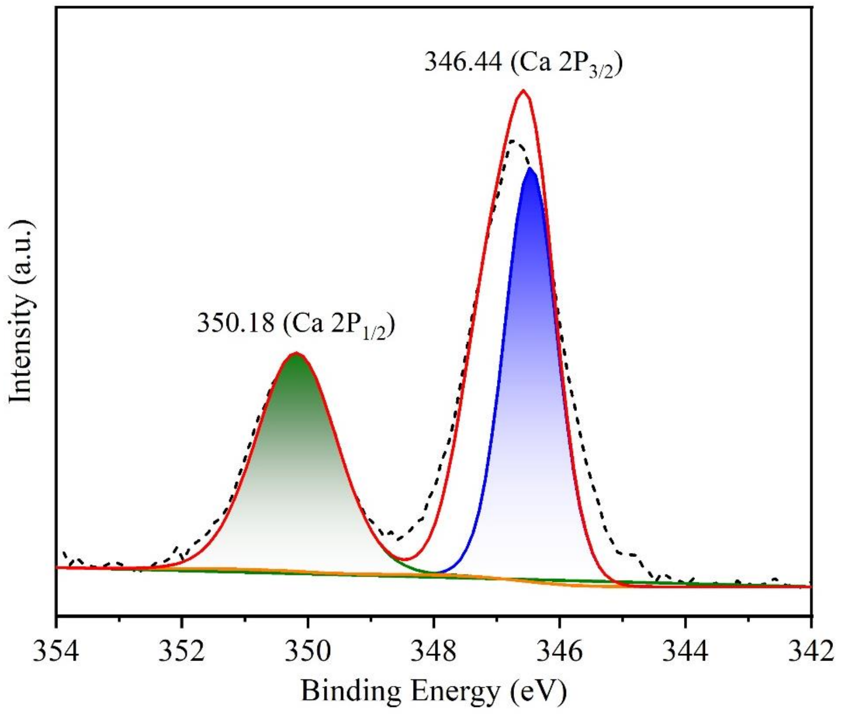

Figure S7. High-resolution spectrum of Ca $2P_{3/2}$ after Pb(II) adsorption.
